# Gene expression analyses determine two different subpopulations in KIT-negative GIST-like (KNGL) patients

**DOI:** 10.18632/oncotarget.24799

**Published:** 2018-04-03

**Authors:** David S. Moura, Rafael Ramos, Antonio Fernandez-Serra, Teresa Serrano, Julia Cruz, Ramiro Alvarez-Alegret, Rosa Ortiz-Duran, Luis Vicioso, Maria Luisa Gomez-Dorronsoro, Xavier Garcia del Muro, Javier Martinez-Trufero, Jordi Rubio-Casadevall, Isabel Sevilla, Nuria Lainez, Antonio Gutierrez, Cesar Serrano, Maria Lopez-Alvarez, Nadia Hindi, Miguel Taron, José Antonio López-Guerrero, Javier Martin-Broto

**Affiliations:** ^1^ Institute of Biomedicine of Sevilla (IBiS, HUVR, CSIC, University of Sevilla), Sevilla, Spain; ^2^ Pathology Department, Son Espases University Hospital, Palma, Illes Baleares, Spain; ^3^ Laboratory of Molecular Biology, Valencian Oncologic Institute, Valencia, Spain; ^4^ Pathology Department, Bellvitge University Hospital, IDIBELL, Barcelona, Spain; ^5^ Pathology Department, Valencian Oncologic Institute, Valencia, Spain; ^6^ Pathology Department, Miguel Servet University Hospital, Zaragoza, Spain; ^7^ Pathology Department, Josep Trueta University Hospital, Girona, Spain; ^8^ Pathology Department, Virgen de la Victoria University Hospital, Malaga, Spain; ^9^ Pathology Department, Hospital Complex of Navarra, Pamplona, Spain; ^10^ Medical Oncology Department, Institut Català d'Oncologia, IDIBELL, Universitat de Barcelona, Barcelona, Spain; ^11^ Medical Oncology Department, Miguel Servet University Hospital, Zaragoza, Spain; ^12^ Medical Oncology Department, Catalan Oncologic Institute, Josep Trueta University Hospital, Girona, Spain; ^13^ Medical Oncology Department, Virgen de la Victoria University Hospital, Malaga, Spain; ^14^ Medical Oncology Department, Hospital Complex of Navarra, Pamplona, Spain; ^15^ Hematology Department, Son Espases University Hospital, Palma, Illes Baleares, Spain; ^16^ Medical Oncology Department, Vall d'Hebron Institute of Oncology, Vall d'Hebron University Hospital, Barcelona, Spain; ^17^ Medical Oncology Department, University Hospital Virgen del Rocio, Sevilla, Spain

**Keywords:** KNGL, miRNA221/222 cluster, KIT, DOG1, IGF1R

## Abstract

**Introduction:**

There are limited findings available on KIT-negative GIST-like (KNGL) population. Also, KIT expression may be post-transcriptionally regulated by miRNA221 and miRNA222. Hence, the aim of this study is to characterize KNGL population, by differential gene expression, and to analyze miRNA221/222 expression and their prognostic value in KNGL patients.

**Methods:**

*KIT*, *PDGFRA*, *DOG1*, *IGF1R*, *MIR221* and *MIR222* expression levels were determined by qRT-PCR. We also analyzed *KIT* and *PDGFRA* mutations, DOG1 expression, by immunohistochemistry, along with clinical and pathological data. Disease-free survival (DFS) and overall survival (OS) differences were calculated using Log-rank test.

**Results:**

Hierarchical cluster analyses from gene expression data identified two groups: group I had *KIT*, *DOG1* and *PDGFRA* overexpression and *IGF1R* underexpression and group II had overexpression of *IGF1R* and low expression of *KIT*, *DOG1* and *PDGFRA*. Group II had a significant worse OS (*p =* 0.013) in all the series, and showed a tendency for worse OS (*p =* 0.11), when analyzed only the localized cases. MiRNA222 expression was significantly lower in a control subset of KIT-positive GIST (*p <* 0.001). OS was significantly worse in KNGL cases with higher expression of *MIR221* (*p =* 0.028) or *MIR222* (*p =* 0.014).

**Conclusions:**

We identified two distinct KNGL subsets, with a different prognostic value. Increased levels of miRNA221/222, which are associated with worse OS, could explain the absence of KIT protein expression of most KNGL tumors.

## INTRODUCTION

Gastrointestinal stromal tumors (GIST) constitute the most frequent type of digestive tract sarcoma, with an incidence of 10 to 14 cases per million people, each year [1–3]. These tumours have mesenchymal origin and represent a morphological and biological continuum, from incidentally discovered microGIST (<10 mm) to clinically detectable GIST [4]. Nonetheless and despite clinico-pathological distinct features, most GISTs share similar precocious genetic alterations, including *KIT* or *PDGFRA* gain-of-function driver mutations [5]. Indeed, numerous studies have confirmed that most GISTs harbour *KIT* (60–85% of cases) or *PDGFRA* (5%–15%) mutations. Additionally, between 10 to 15% of GISTs lack detectable mutations in both receptors, being these cases considered GIST WT for *KIT* and *PDGFRA* [6]. Accordingly, it seems reasonable that other unknown or barely characterized molecular pathways may be implicated in GIST development [7–10]. The description of new potential therapeutic targets in KIT-negative GIST-like (KNGL), might lead to novel molecular targeted therapies, as it is applied nowadays in *KIT*/ *PDGFRA*-driven GISTs [5, 11].

The positive immunostaining of KIT (CD117) has been a pathologic cornerstone for GIST diagnosis, since its redefinition in 2001 [12, 13]. Apart from mutations in disease-driven *KIT* gene, the regulatory molecular mechanisms of KIT expression in GISTs are not yet fully understood. The overexpression of KIT is rarely due to gene amplification, but may be related to dysregulation of *KIT* gene transcription [14]. Nonetheless, less than 5% of these cases show histopathological features of GIST, but with negative or very weak immunohistochemical KIT protein expression - KNGL tumors [15]. Within KNGL tumors two different subgroups were described according to pathologic and molecular findings: 1) DOG1 positive cases, which are predominantly gastric and carry more frequently *PDGFRA* mutations; and 2) DOG1 negative cases, that are either gastric or intestinal and with predominant WT genotype [16, 17].

Gene expression is also regulated by epigenetic mechanisms such as microRNAs (miRNAs). MiRNAs are small (17–27 nt) non-coding single stranded RNA molecules that negatively regulate gene expression by binding to imperfect complementary sites within the 3′ untranslated region (UTR) of their mRNA target at the post-transcriptional level [18]. In this sense, miRNA221/222 cluster members have been described to be relevant in the regulation of KIT expression [19]. Bioinformatic analysis suggested that miRNA221 and miRNA222 targeted the 3′ UTR of *KIT* mRNA, downregulating KIT protein expression, which inhibited normal erythropoiesis and erythroleukemic cell growth [19]. Based on these observations our group had shown the prognostic relevance of these miRNAs, in a KNGL series, as well as, its correlation with KIT expression [20]. At the same time, Koelz and colleagues published in 2011, that miRNA221/222 expression levels were significantly overexpressed in KNGL, indicating both publications that this miRNA cluster acts as a regulator of *KIT* expression. Nevertheless, no correlation was observed between the expression of miRNA221 and miRNA222 and histomorphological parameters, tumor risk grade, or KIT mutation status in the past.

With the aim to better characterize this KNGL patient, an expression analysis of genes involved in both regulation of KIT expression: *MIR221* and *MIR222* [21]; as well as in the pathogenesis of GIST: *KIT, PDGFRA, DOG1* [22, 23] and *IGF1R* and *KDR* [24, 25], along with a KIT and PDGFRA mutational screening and pathologic review was performed. The prognostic impact of these alterations was also analyzed.

## RESULTS

### Demographics and pathologic features

A cohort of 33 (5.2%) KNGL cases, derived from a double-blinded reviewed collection of 624 GIST and that were included in the on-line Registry of the Spanish Group for Research on Sarcomas (GEIS), was evaluated in this study. One case was dropped out because the patient was diagnosed with GIST and died after undergoing to urgent laparotomy due to acute peritonitis secondary to gastric perforation. The male: female ratio was 1:1 with a median age of 61 years-old (range: 25–74). All patients were diagnosed between 1992 and 2003 and GIST morphology validated by two independent expert pathologists.

Median follow-up was of 92 months and a total of 14 events of metastases were registered. Five cases were metastatic at diagnosis. Median size of primary tumours was 5 cm (ranging from 0.5 to 20 cm) and primary tumour locations were stomach (*n =* 15), small-bowel (*n =* 10), rectum (*n =* 4), colon (*n =* 1) and omentum (*n =* 2). Most of the patients (84.4%) were symptomatic at the time of diagnosis and the main symptoms leading to diagnosis was distributed as follows: constitutional syndrome related with anemia (5 pts), high digestive hemorrhage and gastrointestinal perforation (7 pts), abdominal pain (11 pts) and others (4 pts). The median mitotic count per 50 high-power field (HPF) was 5.5 (range 0-200/ HPF) and among the 32 cases analyzed, 14 showed an epithelioid histology (43.8%), 5 spindle cell (15.6%), 5 mixed spindle cell and epithelioid (15.6%), and 8 showed pleomorphic histology (25.0%). Of 32 cases 20 patients (62.5%) showed negative KIT expression, whereas 12 subjects (37.5%) displayed very weak KIT expression (≤ 10% of cells express KIT) (Supplementary Figure 1). All the cases included were tested for KIT in more than one paraffin block (median 60% of all tumour; range 16%-100%), avoiding the potential heterogeneity of KIT expression in GIST samples. Demographic, clinical and pathologic characteristics of KNGL cases are represented in Table 1.

**Table 1:** Demographics and clinical-pathologic information

**Age**: median (range)	61 (25–74)
**Sex**: male/female (%)	16 (50%)/16 (50%)
**Median follow-up** (months)	92
**Median size of primary tumors** (cm; range)	5 (0.5–20)
**Median mitotic count (/50HPF)** (range)	5.5 (0–200)
**Primary tumor presentation:**	
Localized (%)	27 (84%)
Metastatic (%)	5 (16%)
Primary tumor site:	
Stomach (%)	15 (47%)
Small-bowel (%)	10 (31%)
Rectum (%)	4 (13%)
Colon (%)	1 (3%)
Omentum (%)	2 (6%)
**Metastatic events** (%)	14 (44%)
**Subtype**:	
Epithelioid (%)	14 (44%)
Others (%)	18 (56%)
**KIT Expression**:	
≤10%	12 (37.5%)
Absolute negative	20 (62.5%)
**Genotype**:	
Wild type	18 (56%)
*KIT* mutation	7 (22%)
*PDGFRA* mutation	7 (22%)

### Genotype

Mutational analysis is displayed in Table 1. No *KIT* and *PDGFRA* mutations were detected in 18 (56%) cases (WT). Seven patients (22%) displayed a *KIT* mutation, being 4 in exon 11: g.558L>R missense mutation, an interstitial deletion affecting codons from 552 to 559 (g.552_559del), a deletion between codons 559 and 565 (g.559_565del) plus a g.558L>N missense mutation, and a g.628S>N missense mutation; one in exon 13, another in exon 17 and other, a complex mutation involving part of intron 11. Moreover, 7 KNGL (22%) showed a *PDGFRA* mutation, six in *PDGFRA* exon 18 (two g842D>V and one g.842D>Y kinase domain mutations, one deletion 848-851 (g.848_851del) plus a missense mutation g.852D>E, one deletion 849-852 (g.849_852del), and one g.827A>T missense mutation) and 1 case in exon 12 (g.561V>D juxtamembrane domain mutation).

Eighteen cases were negative for *KIT* and *PDGFRA* mutations and among them 5 cases with pleomorphic histology and 2 with fusocellular histology displayed absolute negative expression of KIT and DOG1. Additional molecular and clinical review was performed to discard dedifferentiated liposarcoma (pleomorphic histology) and desmoid tumor (fusocellular histology) in these cases. Amplification of *MDM2* has been described in a small percentage of GIST cases (3.0% to 5.3%) and it is associated with clinical and histological malignancy in this population [26, 27]. Notwithstanding, we tested all the 5 pleomorphic cases for *MDM2* amplification and expression, by FISH and qRT-PCR, respectively (Supplementary Figure 2). Amplification of *MDM2* was detected in one case, by FISH, and by qRT-PCR, the same case showed moderate expression, compared with a dedifferentiated liposarcoma positive control, of both *MDM2* and *CDK4*, a gene well known to be amplified in dedifferentiated liposarcoma and described to be not amplified in GIST [27]. Clinical review discarded dedifferentiated liposarcoma of this case. Neither the radiological appearance, nor the tumor location indicated a dedifferentiated liposarcoma. The intra-peritoneal primary location (large intestine) is not associated with primary dedifferentiated liposarcoma and the typical transition between the well differentiated (lipomatous) and dedifferentiated areas was not detected neither in CAT scan nor in histological review of the tumor. On the other hand, desmoid tumor was discarded in both fusocellular histology cases. These cases showed cytoplasmic β-catenin location and it was not detected any mutation in the *CTNNB1* gene (Supplementary Figure 3).

### Survival analysis

Twenty-seven KNGL patients, with localized disease at diagnosis, were included in the univariate analysis with a median follow-up of 92 months (Table 2). Gastric location of the primary tumor versus other locations (*p =* 0.019) and *PDGFRA* mutations versus WT or *KIT* mutated (*p =* 0.043) were related with a significantly better disease-free survival (DFS). No differences were observed for DFS at seven years, among the sub-groups concerning cellularity, size, and number of mitotic figures, mutations in *KIT* exon 11 and histology. Moreover, tumors were assigned into risk groups according to their pathologic features. No differences were detected in DFS for Miettinen or Fletcher’s risk groups. On the other hand, two factors significantly influenced a worse OS: non-gastric location (*p =* 0.036), age higher than 60 years (*p =* 0.03).

**Table 2:** Univariate Survival Analysis^1^

Variable	DFS at 7 years (95% CI)	*p*	OS at 7 years (95% CI)	*p*
**Miettinen**:		0.21		0.49
Low (*n =* 13)	67% (35–98)		64% (36–93)	
Intermediate (*n =* 3)	100%		100%	
High (*n =* 11)	54% (25–84)		70% (42–98)	
**Fletcher**:		0.23		0.34
Low (*n =* 11)	56% (17–95)		70% (41–999)	
Intermediate (*n =* 4)	100%		100%	
High (*n =* 12)	58% (30–86)		73% (46–99)	
**Cellularity**:		0.24		0.47
Normal (*n =* 17)	76% (52–100)		75% (53–96)	
High (*n =* 10)	50% (19–81)		55% (24–82)	
**Size (cm)**:		0.82		0.85
0-6 (*n =* 16)	66% (38–94)		73% (51–96)	
6-10 (*n =* 2)	67% (13–100)		67% (13–100)	
>10 (*n =* 9)	62% (29–96)		86% (60–100)	
**Number of Mitosis**:		0.064		0.2
0-10 MPF (*n =* 18)	79% (57–100)		82% (64–100)	
>10 MPF (*n =* 9)	44% (12–77)		62% (29–96)	
**Location**:		0.019		0.036
Gastric (*n =* 15)	83% (61–100)		91% (74–100)	
Others (*n =* 12)	45% (16–75)		64% (36–92)	
**Age**:		0.39		0.03
0-60 years (*n =* 14)	70% (45–95)		92% (78–100)	
>60 years (*n =* 12)	56% (24–89)		55% (24–85)	
**Diagnostic Delay (Months)**:		0.35		0.85
0-1 (*n =* 14)	61% (36–86)		82% (63–100)	
>1 (*n =* 13)	75% (45–100)		67% (36–97)	
**KIT Exon 11 Mutated**^**2**^:		0.88		0.71
No (*n =* 24)	50% (1–99)		75% (32–100)	
Yes (*n =* 3)	65% (43–88)		77% (58–97)	
**KIT Expression**:		0.52		0.3
≤10% (*n =* 16)	68% (37–99)		81% (57–100)	
Absolute negative (*n =* 11)	62% (36–89)		73% (51–96)	
**Subtype**:		0.72		0.15
Epithelioid (*n =* 14)	69% (44–95)		92% (78–100)	
Others (*n =* 13)	61% (31–91)		57% (28–86)	
**Expression group**^**3**^:		0.19		0.11
I (*n =* 13)	83% (62–100)		82% (59–100)	
II (*n =* 14)	49% (20–78)		70% (45–95)	
**Mutations**:		0.043		0.45
*PDGFRA* (*n =* 7)	100%		100%	
Others (WT and *KIT*) (*n =* 20)	54% (31–78)		76% (57–94)	

^1^Localized disease at diagnosis; ^2^ Mutations affecting codon 557 and/ or 558 ^3^I – *KIT*/ *PDGFR*/ *DOG1* positive and II – *IGF1R* positive

### Gene expression analysis

All the 32 cases were included in gene expression analysis. For gene expression analyses, five genes were selected based on their relevance on GIST pathology (i.e. *KIT*, *PDGFRA* and *DOG1*) or due to their importance in angiogenesis and the reasonable efficiency of anti-angiogenic therapy, such as pazopanib and sunitinib, in GIST (i.e. *IGF1R* and *KDR*) [28].

**Figure 1:**
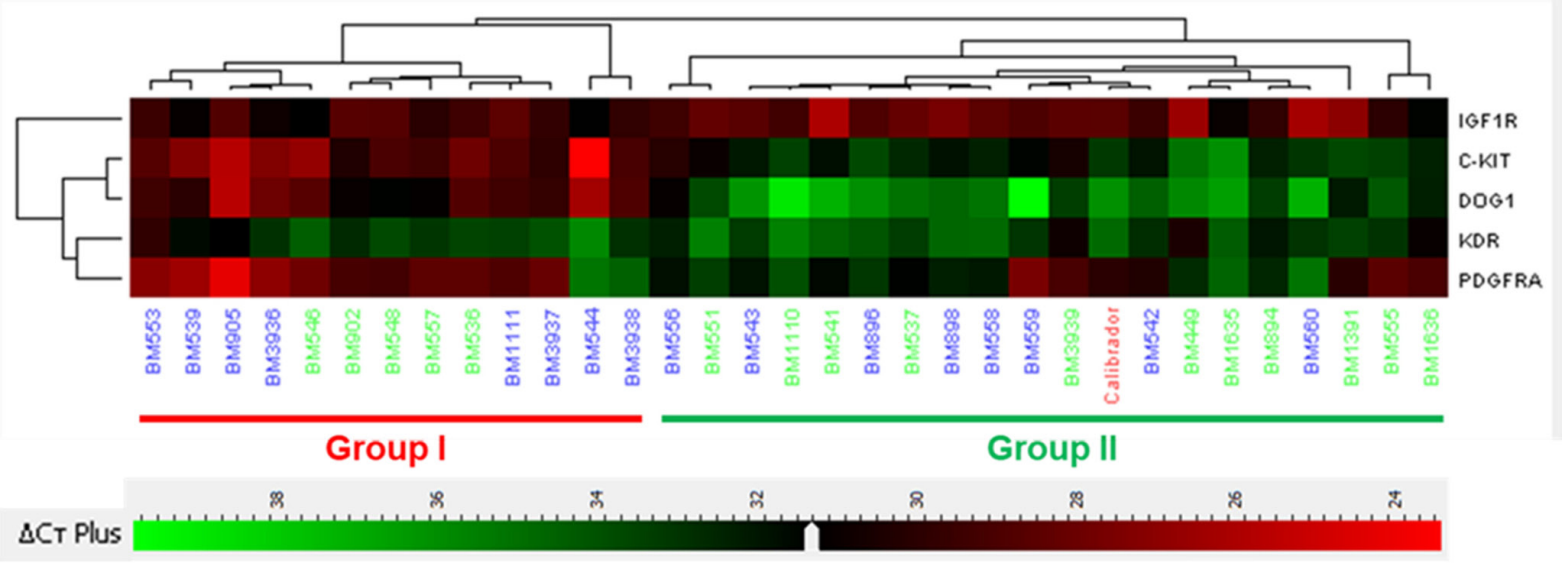
Clustering analyses according to RNA level expression Group I (13 cases) is characterized by an overexpression of *KIT*, *DOG1* and *PDGFRA*, and underexpression of *IGF1R*. Group II (19 cases) is characterized by an overexpression of *IGF1R*. In this group *KIT*, *DOG1* and PDGFRA are clearly underexpressed. KDR expression was similar between both groups. Numbers in green indicate wild type genotype.

**Figure 2:**
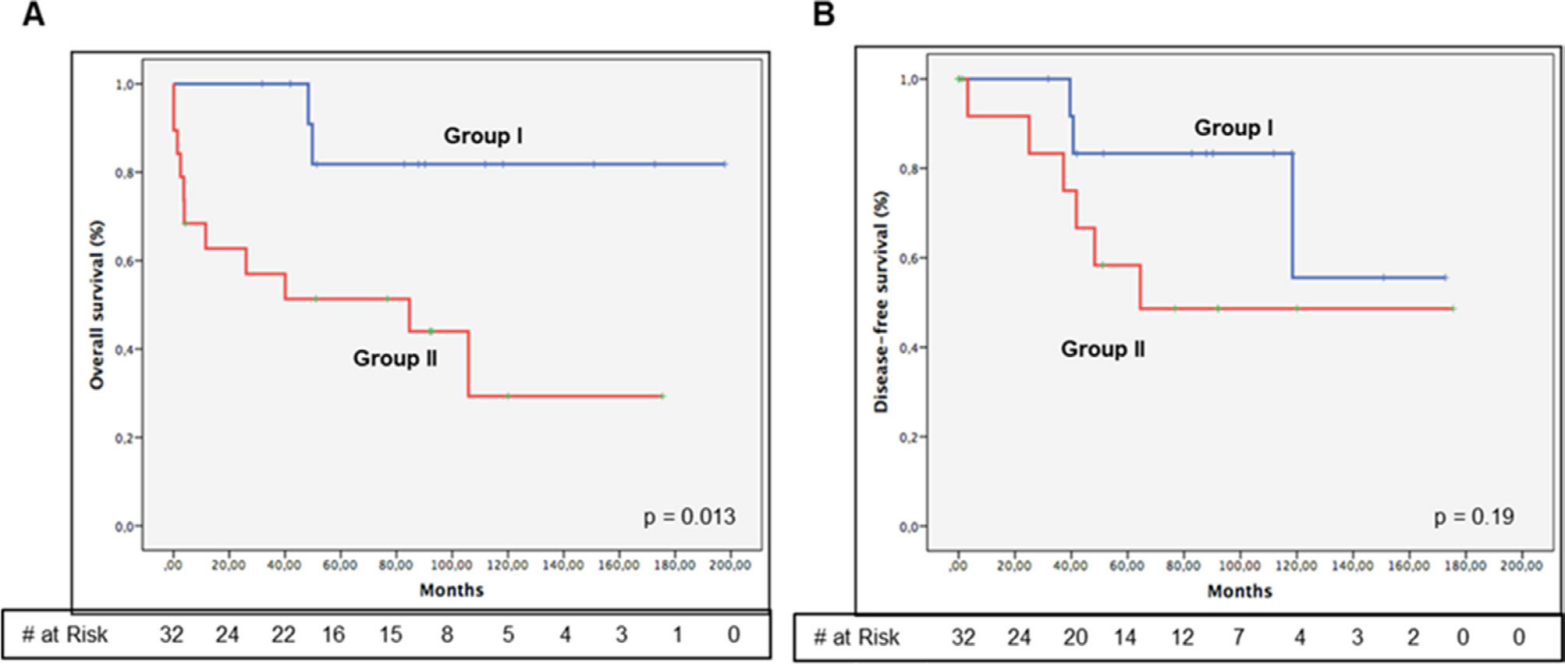
Kaplan-Meyer estimation of overall survival (**A**) and disease-free survival (**B**) between gene expression groups. Group I show overexpression of *KIT*, *DOG1* and *PDGFRA*, and low levels of *IGF1R*. Group II shows an inversed gene expression pattern, overexpressing *IGF1R* and underexpressing *KIT*, *DOG1* and *PDGFRA*. Group I has better overall survival (*p* = 0.013), compared with Group II.

Unsupervised hierarchical cluster analyses obtained from KIT, PDGFRA, DOG1, IGF1R and KDR gene expression data identified two distinct groups (Figure 1): Group I (13 patients) had overexpression of KIT (23.46 median fold), DOG1 (111.97 median fold) and *PDGFRA* (2.96 median fold), and underexpression of *IGF1R* (0.44 median fold); Group I cases had predominance of epithelioid features, gastric location, DOG1 positive immunostaining (Supplementary Figure 1) and PDGFRA mutations. Group II (19 patients) had a higher expression of IGF1R (0.98 median fold), and a lower expression of KIT (1.76 median fold), DOG1 (2.02 median fold) and PDGFRA (0.26 median fold); most cases were non-gastric with DOG1 negative immunostaining and *KIT* mutated or WT. Expression fold of *KDR* was similar among both groups (3.39 median fold in Group I and 2.96 median fold in Group II). Group I and II characteristics are compared in Table 3. Analysis of survival between the two expression groups showed that Group II had significant worse prognosis for OS (*p =* 0.013), but not for DFS (0.19), compared to Group I (Figure 2). Survival analysis taking into consideration only the sub-group of *KIT* and *PDGFRA* mutated cases showed a trend to a worse OS (*p =* 0.123) and DFS (*p =* 0.099) (Supplementary Figure 4) of Group II compared to Group I.

**Table 3:** Differences in pathologic, mutational and mRNA levels in gene expression groups

Groups	Location	Cell type	DOG1 staining	Mutational state	*KIT* Expression
**I** (*n =* 13)	**Gastric:** 11	**Epithelioid:** 10	**Positive:** 9	***PDGFRA***: 6	: 23.46
	**Others:** 2	**Others:** 3	**Negative:** 0	***KIT*:** 2	**High**: 13/13
			**N/A:** 4	**Wild Type:** 5	**Low**: 0/13
**II** (*n =* 19)	**Gastric:** 4	**Epithelioid:** 4	**Positive:** 1	***PDGFRA***: 1	: 1.76
	**Others:** 15	**Others:** 15	**Negative:** 17	***KIT*:** 5	**High**: 3/19
			**N/A:** 1	**Wild Type:** 13	**Low**: 16/19
					*p <* 0.001
Groups	*PDGFRA*	*DOG1*	*IGF1R*	*MIR221*	*MIR222*
	Expression	Expression	Expression	Expression	Expression
**I** (*n =* 13)	$\tilde{X}$: 2.96	$\tilde{X}$: 111.97	$\tilde{X}$: 0.44	$\tilde{X}$: 0.02	$\tilde{X}$: 3.16
	**High**: 11/13	**High**: 13/13	**High**: 4/13	**High**: 4/11	**High**: 2/10
	**Low**: 2/13	**Low**: 0/13	**Low**: 9/13	**Low**: 7/11	**Low**: 8/10
**II** (*n =* 19)	$\tilde{X}$: 0.26	$\tilde{X}$: 2.02	$\tilde{X}$: 0.98	$\tilde{X}$: 0.09	$\tilde{X}$: 9.14
	**High**: 5/19	**High**: 3/19	**High**: 12/19	**High**: 11/18	**High**: 11/17
	**Low**: 14/19	**Low**: 16/19	**Low**: 7/19	**Low**: 7/18	**Low**: 6/17
	*p =* 0.005	*p <* 0.001	*p =* 0.018	*p =* 0.15	*p =* 0.11

N/A: Not Available. $\tilde{X}$: Median.

### MiRNA221 and miRNA222 expression

**Figure 3:**
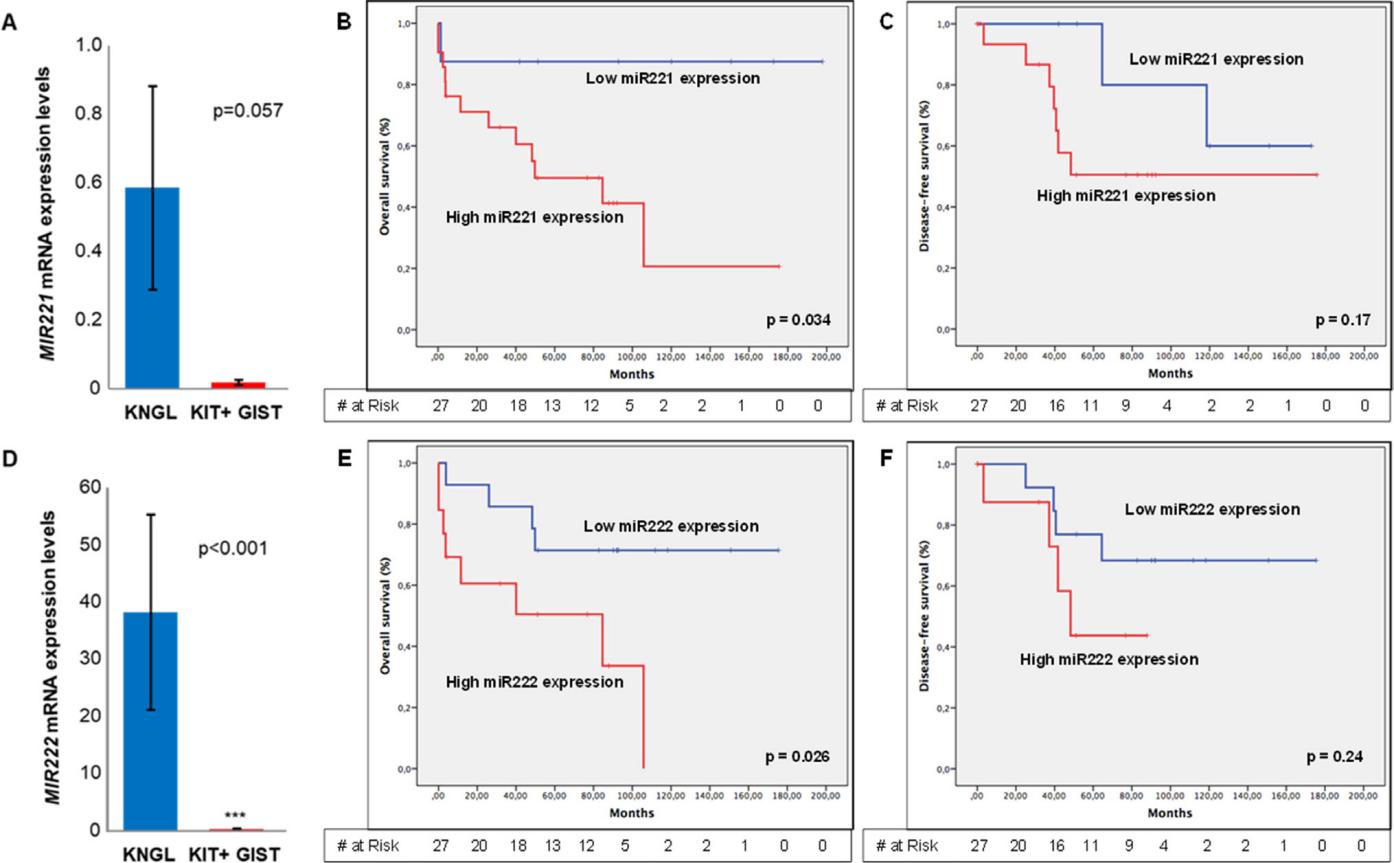
*MIR212* and *MIR222* expression levels and Kaplan-Meyer estimation of overall or disease-free survival, regarding miRNAs expression levels (**A**) Absolute expression levels of *MIR221* in KNGL and KIT positive GIST cases (positive control). (**B**) Overall survival and (**C**) Disease free survival considering MIR221 gene expression. (**D**) Absolute expression levels of *MIR222* in KNGL and KIT positive GIST cases. (**E**) Overall survival and (**F**) Disease-free survival concerning *MIR222* gene expression. Error bar represents standard error. ^*^p ≤ 0.05, ^**^p ≤ 0.005 and ^***^p ≤ 0.0005.

Nine additional KIT positive cases, by immunohistochemistry, were included in the study to compare the miRNAs expression levels with KNGL cases. Expression of both *MIR221* and *MIR222* was similar between gene expression groups I and II (Table 3). However, the expression of *MIR221* and *MIR222* was significantly lower in the KIT positive cases compared with this KNGL series (Figure 3A and 3D, respectively): for miRNA221 being 0.59 in KNGL vs. 0.018 in KIT positive control group, *p =* 0.057; and for miRNA222 38.26 vs. 0.29, *p <* 0.001. Likewise, it seems to be an inverse correlation between *KIT* expression levels and *MIR221* (Spearman’s ρ = –0.447; *p ≤* 0.05) or *MIR222* (Spearman’s ρ = –0.419; *p ≤* 0.05) in KNGL series (Table III). Additionally, a correlation between miRNA222 expression levels and the mutational status of KNGL has also been observed (Supplementary Figure 5), in the sense that, WT tumors show overexpression of *MIR222*, compared with the mutated cases (*p =* 0.034). Interestingly, the OS was significantly worse for GIST cases with higher *MIR221* (*p =* 0.034) or *MIR222* (*p =* 0.026) expression, suggesting a potential prognostic value for these two miRNAs in KNGL population (Figure 3B and 3E, respectively). Yet, no association was detected between DFS and *MIR221* (*p =* 0.17) or *MIR222* (*p =* 0.24) expression in the same series (Figure 3C and 3F, respectively). On the other hand, univariate analysis showed that high expression of *MIR221* was associated with a gastric location (*p =* 0.038) and a higher number of mitosis per 50 HPF (*p =* 0.044), whereas cases with high expression of *MIR222* were related with complete negative expression of KIT (*p =* 0.018). *MIR222* was also significantly expressed in Group II (*p =* 0.046) (Supplementary Table 1).

## DISCUSSION

GISTs represent a solid tumour model for molecular targeted therapy having approved different tyrosine kinase inhibitors for three lines of treatment. Usually, these tumours arise from driver-mutations in *KIT* or *PDGFRA* genes [29] and it is nowadays widely accepted that KIT protein expression is a hallmark of GIST. Nevertheless, less than 5% of GIST cases lack or slightly express KIT protein by immunohistochemistry (IHC) [15, 17] which indicates that some unknown mechanisms may be underlying GIST pathogenesis and/ or regulating KIT expression.

The hierarchical cluster analyses obtained from our KNGL series, distinguished two completely separated groups depending on the expression of *KIT*, *PDGFRA*, *DOG1,* and *IGF1R*. Group I was characterized by the overexpression of *KIT*, *DOG1* and *PDGFRA*, and low expression of *IGF1R*, while Group II showed a completely different expression profile with overexpression of *IGF1R* and low expression of *KIT*, *DOG1* and *PDGFRA. IGF1R* is normally overexpressed in WT GISTs [28, 30], independently of copy number abnormalities or activating mutations [31], and IGF1R pathway seems to be involved in the differentiation of interstitial cells of Cajal (GIST precursor cell) immature into mature interstitial cells of Cajal [32], as well as in GIST pathogenesis and development [25, 32] and imatinib resistance [25]. This could indicate that in our KNGL series, cases overexpressing *IGF1R* may be associated with a more stem-like phenotype. Besides, overexpression of IGF1R and its respective ligands IGF1 and IGF2 seems to be prognostic factors for relapse in operated high-risk GIST patients [33]. Similar results were obtained in our study in the KNGL series, where Group II with *IGF1R* mRNA overexpression was associated with worst OS. Nevertheless, conflicting results had been also published [34, 35]. Yet, it is necessary to point out that our results are based on *IGF1R* genomic analysis, rather than protein evaluation by IHC, which may justify the lack of correlation between expression and survival. Also, it is critical to consider that our series includes only KNGL patients.

Group I cases were predominantly epithelioid gastric cases, with DOG1 positive immunostaining and *PDGFRA* mutations, whereas Group II series were mostly non-gastric with DOG1 negative immunostaining and *KIT* mutated or WT. These results are in line with previous published data, where two distinct subsets of KNGL were described, depending on DOG1 immunostaining. DOG1 positive cases were described to be mainly gastric and associated with PDGFRA mutations and DOG1 negative cases were described to be gastric and/ or intestinal and usually WT [16]. Remarkably, negative DOG-1 protein expression seems to be an independent prognostic factor for shorter OS [34, 36]. Similar results come out in our study, since Group II (negative DOG1 immunostaining) showed worse prognosis for OS. Likewise, GIST histological type has also been described to have a significant impact in GIST patient’s prognosis [37, 38], however with inconsistent results [39, 40]. In a report with 48 patients, the 5-year recurrence-free survival rate was significantly better among patients with spindle cell as compared to epithelioid or mixed histology in a series of KIT-expressing series [37]. Yet, univariate survival analysis showed that within our KNGL series, histological type was not a prognostic biomarker for survival, indicating that both DFS and OS related with GIST histological type may depend on KIT protein expression. Also, Group I features seemed to correspond with the typical features and behaviour of PDGFRA-driven GISTs.

Our results demonstrated that miRNA222 may be relevant in the regulation of KIT protein expression, since it is overexpressed in KNGL series, compared to GIST cases. *MIR222* was significantly overexpressed in our KNGL population, whereas *MIR221* showed a trend for signification. These results are in line with previous published data, wherein it was demonstrated, by luciferase assay, that both miRNA221 and miRNA222 were direct regulators of *KIT* expression, targeting the 3′ UTR of *KIT* mRNA [19, 41], and that the levels of this two miRNAs were significantly lower in KIT-expressing GISTs, compared to KIT-negative GISTs [42, 43]. More importantly, our study demonstrated for the first time, that low levels of either miRNA221 or miRNA222 are relevant prognostic biomarkers for better OS, in KNGL patients. Activating mutations in *KIT* are present in about 80% of GIST [11, 40, 44] and lower expression of miRNA221 and miRNA222 may play a role in further enhancing KIT oncogenic influence on the cell in these cases. Yet, in KNGL cases, the role of KIT in tumour pathogenesis may be limited [10, 45], which indicates that the prognostic value of the miRNAs may also be by targeting other genes, besides KIT. Accordingly, targeting of cell cycle inhibitors *CDKN1B* (p27^Kip1^) and *CDKN1C* (p57^Kip2^) [46–48], or even of the tumour suppressor protein PTEN [49], by miRNA221 and miRNA222 may help understand the worst survival prognosis, when each one of the miRNAs is overexpressed in KNGL context. Besides, it is also important to take notice the association between miRNA221 overexpression and a tendency for a higher number of mitotic figures and, the higher levels of miRNA222 in Group II, overexpressing *IGF1R*. Thus, it seems that overexpression of miRNA221 could be associated with a higher proliferation rate in the KNGL series, whereas higher expression of miRNA222 could be associated with a more aggressive stem-like phenotype, which may justify the worst OS associated with both these miRNAs. On the other hand, the prognostic data of miRNA221/222 cluster members is in some way inconsistent with previous pre-clinical results, where the overexpression of miRNA221 and miRNA222, in GIST cell lines, inhibited cell proliferation, disturbed cell cycle progression and increased apoptosis [41, 50] by a signaling cascade that may involve KIT, AKT and BCL2 [50]. However, these experiments were performed in KIT-mutated GIST cell lines, which differ from our KNGL context, and therefore, it may explain the distinct prognostic value of miRNA221 and miRNA222 in both studies.

Overall, our results described two completely separated groups depending on the expression of 5 genes. Moreover, our work showed that the overexpression of miRNA222, and to a lesser extent of miRNA221, could explain the absence of KIT protein expression in the biggest cohort ever analysed of KNGL cases (*N =* 32), even when *KIT* mRNA is overexpressed. These miRNAs showed in KNGL patients an interesting prognostic profile. Further investigation is necessary to understand the potential targets underlying the worse prognostic value associated with miRNA221/222 cluster members’ expression.

## METHODS

### Patients

Cases selected for this study, from on line GIST Registry of GEIS, had to accomplish with the following criteria: Absence or weak (≤ 10*%*) protein KIT expression, being not received adjuvant imatinib, available paraffin embedded block at diagnostic time and minimum follow-up of 6 months. Clinical and pathological data collected in the study were: age at diagnosis, gender, performance status at diagnosis, tumor location, staging, tumor size, type of surgery, status of surgical margins, data on systemic treatment(s) if any, mitotic count, necrotic extension, tumor rupture, type of cellularity and cellular density, histologic sub-type and cellularity. The status at last follow-up (alive with or without disease, dead with or without disease), date and type of recurrence and any administered systemic treatment were obtained by follow-up from medical oncologists. Postoperative surveillance varied between different centers, but all of them consistently performed CT scan every 4–6 months, during the first 5 years after surgery. Clinical data was updated and checked on a queries-based task between data center and medical oncologists from each of participating centers. Two pathologists (R.R. and J.C.), with expertise in GIST, were in charge of the independent pathologic review of the paraffin-embedded tissue blocks (diagnostic/ surgical specimen). The potential differential diagnosis as lymphoma, neuroectodermal tumors, solitary fibrous tumors or leiomyosarcoma, dedifferentiated liposarcoma or desmoid tumor, was ruled out by both the pathologists.

### Immunohistochemistry

KIT and DOG1 were selected for protein expression analysis by IHC. For all these biomarkers, correlation to clinical outcome was carried out. IHC studies were performed on formalin-fixed, paraffin-embedded tissues (FFPE) in 3- to 4-μm sections, using the following antibodies: KIT [40] (1:400; A-4502; DAKO; Copenhagen, Denmark), DOG1 [16] (1:75; Clone K9, Novocastra, Leica Biosystems, Wetzlar, Germany) and β-catenin (610153; BD Biosciences, Qume Drive, San Jose, CA, USA). For KNGL classification, pathologist required negative o very weak KIT expression (≤ 10% of cells express KIT).

### Fluorescence in situ hybridization (FISH)

Histological sections of FFPE tissues were deparaffinized and hydrated in alcohols of decreasing concentrations. Antigenic recovery was performed with citrate at pH6. The tissue sections were co-denatured with proteinase K and the Vysis LSI MDM2 SO/CEP12 FISH probe (Abbott Molecular; E Touhy Ave, Des Plaines, IL, USA), at 90° C for 5 minutes, and the hybridization was done overnight at 37° C. After hybridization, the slides were washed with a 2X SSC/Tween20 solution, the nuclei counterstained with DAPI II (Abbott Molecular) and, the results were visualized in a fluorescence microscope equipped with the appropriate filters and a digital camera (Leica).

### Genotype

DNA was isolated from 3- to 5-μm sections of FFPE tissues. After deparaffinization, the tumor tissue was processed with the QIAamp DNA Investigator Kit (Qiagen, Valencia, CA) according to the manufacturer´s instructions. Intronic primers were used to amplify exons 9, 11, 13 [51] and 17 [52] of *KIT,* exons 12 and 18 of *PDGFRA* [5] and exon 3 of *CTNNB1* [53] by PCR in a reaction volume of 25 µL containing 5 µL of DNA, with 2,5 µL of buffer and MgCl_2_ 25 mM and 0.5 µL of dNTP and 1.5 Units of ampliTag gold (Applied Biosystems, Foster City, Ca). PCR products were sequenced in F and R, after preheating the samples at 94° C for 6 minutes. The DNA was amplified over 40 cycles of 45 seconds of denaturation at 94° C; 1 minutes of annealing at 56° C and 1 minute of extension at 72° C, with an additional final extension step of 10 minutes. Negative controls were included in every set of amplifications. Bidirectional sequencing with specific primers was performed in an ABI 3130 xl genetic analyzer using the BigDye Terminator v3.1 kit (Applied Biosystems).

### Gene expression analyses

The gene expression of *KIT*, *PDGFRA*, *DOG1, KDR, IGF1R, MIR221* and *MIR222* was determined by qRT-PCR, using total RNA extracted, from three 20-µm thick FFPE section, with the Recover All Total Nucleic Acid Isolation^®^ (Ambion, Austin, USA). For the expression analysis, the following TaqMan assays were used: *KIT (*Hs00174029_m1*), PDGFRA (*Hs00998018_m1*), DOG1 (*Hs00216121_m1*), IGF1R* (Hs00609576_m1), *KDR* (VEGFR2) (Hs00911700_m1), *MIR-221* (Hs04231481_s1) and *MIR-222* (Hs04415495_s1), and *MDM2* (Hs1066930_m1) and *CDK4* (Hs01565683_g1). The qPCR was performed in a 7500 Fast Real Time PCR system (Applied Biosystems, Foster City, CA), with specific protocols for both quantification of mRNA and miRNA according to manufacturer´s instructions. Relative expression was calculated using the comparative Ct method (2^−ΔΔCt^) against universal human reference RNA (RNA pool: Universal Human Reference RNA; Agilent Technologies, Santa Clara, CA, USA). Nine KIT positive GISTs were included in gene expression analyses to compare miRNAs levels with KNGL cases.

### Statistical analysis

Categorical variables were reported as relative frequencies (%). Quantitative variables were expressed as median and ranges. Comparisons between categorical variables and qualitative variables were performed with the Chi-square or Fisher exact test when appropriated, while in the case of quantitative variables the non-parametric *U* de Mann–Whitney test was used. OS and DFS were measured from the date of diagnosis to the final event, and were estimated according to the Kaplan-Meier method. Comparisons between the variables of interest were performed by the log-rank test. To analyze the impact on survival of *MIR221* and *MIR222*, these variables were stratified as high or low expression groups according to the cutoff obtained using Receiver Operating Characteristics (ROC) curves. All *p* values reported were 2-sided, and statistical significance was defined at *p =* 0.05. All the statistical procedures were performed with SPSS 20.0 software.

## SUPPLEMENTARY MATERIALS FIGURES AND TABLE



## Abbreviations

DFS: Disease-Free Survival
FFPE: Formalin- Fixed, Paraffin-Embedded
GIST: Gastrointestinal Stromal Tumor
IHC: Immunohistochemistry
KNGL: KIT-Negative GIST-Like
MPF: Mitosis per Field
OS: Overall Survival
UTR: Untranslated Region
WT: Wild Type

## References

[1] Rubio J, Marcos-Gragera R, Ortiz MR, Miro J, Vilardell L, Girones J, Hernandez-Yague X, Codina-Cazador A, Bernado L, Izquierdo A, Colomer R (2007). Population-based incidence and survival of gastrointestinal stromal tumours (GIST) in Girona, Spain. Eur J Cancer.

[2] Joensuu H (2006). Gastrointestinal stromal tumor (GIST). Ann Oncol.

[3] Soreide K, Sandvik OM, Soreide JA, Giljaca V, Jureckova A, Bulusu VR (2016). Global epidemiology of gastrointestinal stromal tumours (GIST): A systematic review of population-based cohort studies. Cancer Epidemiol.

[4] Miettinen M, Lasota J (2006). Pathology and prognosis of gastrointestinal stromal tumors at different sites. Semin Diagn Pathol.

[5] Heinrich MC, Corless CL, Duensing A, McGreevey L, Chen CJ, Joseph N, Singer S, Griffith DJ, Haley A, Town A, Demetri GD, Fletcher CD, Fletcher JA (2003). PDGFRA activating mutations in gastrointestinal stromal tumors. Science.

[6] Nannini M, Astolfi A, Urbini M, Indio V, Santini D, Heinrich MC, Corless CL, Ceccarelli C, Saponara M, Mandrioli A, Lolli C, Ercolani G, Brandi G (2014). Integrated genomic study of quadruple-WT GIST (KIT/PDGFRA/SDH/RAS pathway wild-type GIST). BMC Cancer.

[7] Janeway KA, Kim SY, Lodish M, Nosé V, Rustin P, Gaal J, Dahia PL, Liegl B, Ball ER, Raygada M, Lai AH, Kelly L, Hornick JL, NIH Pediatric and Wild-Type GIST Clinic (2011). Defects in succinate dehydrogenase in gastrointestinal stromal tumors lacking KIT and PDGFRA mutations. Proc Natl Acad Sci U S A.

[8] Janeway KA, Zhu MJ, Barretina J, Perez-Atayde A, Demetri GD, Fletcher JA (2010). Strong expression of IGF1R in pediatric gastrointestinal stromal tumors without IGF1R genomic amplification. Int J Cancer.

[9] Pantaleo MA, Urbini M, Indio V, Ravegnini G, Nannini M, De Luca M, Tarantino G, Angelini S, Gronchi A, Vincenzi B, Grignani G, Colombo C, Fumagalli E (2017). Genome-wide Analyses Identifies MEN1 and MAX Mutations and a Neuroendocrine-like Molecular Heterogeneity in Quadruple WT GIST. Mol Cancer Res.

[10] Shi E, Chmielecki J, Tang CM, Wang K, Heinrich MC, Kang G, Corless CL, Hong D, Fero KE, Murphy JD, Fanta PT, Ali SM, De Siena M (2016). FGFR1 and NTRK3 actionable alterations in “Wild-Type” gastrointestinal stromal tumors. J Transl Med.

[11] Martin-Broto J, Rubio L, Alemany R, Lopez-Guerrero JA (2010). Clinical implications of KIT and PDGFRA genotyping in GIST. Clin Transl Oncol.

[12] Fletcher CD, Berman JJ, Corless C, Gorstein F, Lasota J, Longley BJ, Miettinen M, O’Leary TJ, Remotti H, Rubin BP, Shmookler B, Sobin LH, Weiss SW (2002). Diagnosis of gastrointestinal stromal tumors: A consensus approach. Hum Pathol.

[13] Miettinen M, Sobin LH (2001). Gastrointestinal stromal tumors in the appendix: a clinicopathologic and immunohistochemical study of four cases. Am J Surg Pathol.

[14] Tabone S, Theou N, Wozniak A, Saffroy R, Deville L, Julie C, Callard P, Lavergne-Slove A, Debiec-Rychter M, Lemoine A, Emile JF (2005). KIT overexpression and amplification in gastrointestinal stromal tumors (GISTs). Biochim Biophys Acta.

[15] Medeiros F, Corless CL, Duensing A, Hornick JL, Oliveira AM, Heinrich MC, Fletcher JA, Fletcher CD (2004). KIT-negative gastrointestinal stromal tumors: proof of concept and therapeutic implications. Am J Surg Pathol.

[16] Miettinen M, Wang ZF, Lasota J (2009). DOG1 antibody in the differential diagnosis of gastrointestinal stromal tumors: a study of 1840 cases. Am J Surg Pathol.

[17] Seo HS, Hyeon JY, Shin OR, Lee HH (2015). C-Kit-Negative Gastrointestinal Stromal Tumor in the Stomach. J Gastric Cancer.

[18] Ryan BM, Robles AI, Harris CC (2010). Genetic variation in microRNA networks: the implications for cancer research. Nat Rev Cancer.

[19] Felli N, Fontana L, Pelosi E, Botta R, Bonci D, Facchiano F, Liuzzi F, Lulli V, Morsilli O, Santoro S, Valtieri M, Calin GA, Liu CG (2005). MicroRNAs 221 and 222 inhibit normal erythropoiesis and erythroleukemic cell growth via kit receptor down-modulation. Proc Natl Acad Sci U S A.

[20] Martin-Broto J, Garcia del Muro X, Gutierrez A, Martinez-Trufero T, Serrano T, Rubió J, Lainez N, Sevilla I, Cruz J, Ramos R, Ortega L, Poveda A, Ramirez M (2011). KIT, DOG1, PDGFR, and IGFR1 gene expression analyses determine two different subpopulations in KIT-negative GIST-like (KNGL) patients. J Clin Oncol.

[21] Brioschi M, Fischer J, Cairoli R, Rossetti S, Pezzetti L, Nichelatti M, Turrini M, Corlazzoli F, Scarpati B, Morra E, Sacchi N, Beghini A (2010). Down-regulation of microRNAs 222/221 in acute myelogenous leukemia with deranged core-binding factor subunits. Neoplasia.

[22] West RB, Corless CL, Chen X, Rubin BP, Subramanian S, Montgomery K, Zhu S, Ball CA, Nielsen TO, Patel R, Goldblum JR, Brown PO, Heinrich MC (2004). The novel marker, DOG1, is expressed ubiquitously in gastrointestinal stromal tumors irrespective of KIT or PDGFRA mutation status. Am J Pathol.

[23] Miwa S, Nakajima T, Murai Y, Takano Y, Sugiyama T (2008). Mutation assay of the novel gene DOG1 in gastrointestinal stromal tumors (GISTs). J Gastroenterol.

[24] Trent JC, Ramdas L, Dupart J, Hunt K, Macapinlac H, Taylor E, Hu L, Salvado A, Abbruzzese JL, Pollock R, Benjamin RS, Zhang W (2006). Early effects of imatinib mesylate on the expression of insulin-like growth factor binding protein-3 and positron emission tomography in patients with gastrointestinal stromal tumor. Cancer.

[25] Tarn C, Rink L, Merkel E, Flieder D, Pathak H, Koumbi D, Testa JR, Eisenberg B, von Mehren M, Godwin AK (2008). Insulin-like growth factor 1 receptor is a potential therapeutic target for gastrointestinal stromal tumors. Proc Natl Acad Sci U S A.

[26] Wallander ML, Layfield LJ, Tripp SR, Schmidt RL (2013). Gastrointestinal stromal tumors: clinical significance of p53 expression, MDM2 amplification, and KIT mutation status. Appl Immunohistochem Mol Morphol.

[27] Tornillo L, Duchini G, Carafa V, Lugli A, Dirnhofer S, Di Vizio D, Boscaino A, Russo R, Tapia C, Schneider-Stock R, Sauter G, Insabato L, Terracciano LM (2005). Patterns of gene amplification in gastrointestinal stromal tumors (GIST). Lab Invest.

[28] Corless CL, Barnett CM, Heinrich MC (2011). Gastrointestinal stromal tumours: origin and molecular oncology. Nat Rev Cancer.

[29] Lasota J, Miettinen M (2006). KIT and PDGFRA mutations in gastrointestinal stromal tumors (GISTs). Semin Diagn Pathol.

[30] Pantaleo MA, Astolfi A, Di Battista M, Heinrich MC, Paterini P, Scotlandi K, Santini D, Catena F, Manara MC, Nannini M, Maleddu A, Saponara M, Lolli C (2009). Insulin-like growth factor 1 receptor expression in wild-type GISTs: a potential novel therapeutic target. Int J Cancer.

[31] Italiano A, Chen J, Zhang L, Hajdu M, Singer S, DeMatteo RP, Antonescu CR (2012). Patterns of deregulation of insulin growth factor signalling pathway in paediatric and adult gastrointestinal stromal tumours. Eur J Cancer.

[32] Lorincz A, Redelman D, Horvath VJ, Bardsley MR, Chen H, Ordog T (2008). Progenitors of interstitial cells of cajal in the postnatal murine stomach. Gastroenterology.

[33] Braconi C, Bracci R, Bearzi I, Bianchi F, Sabato S, Mandolesi A, Belvederesi L, Cascinu S, Valeri N, Cellerino R (2008). Insulin-like growth factor (IGF) 1 and 2 help to predict disease outcome in GIST patients. Ann Oncol.

[34] Kisluk J, Zinczuk J, Kemona A, Guzinska-Ustymowicz K, Zurawska J, Kedra B (2016). Expression of CD117, DOG-1, and IGF-1R in gastrointestinal stromal tumours - an analysis of 70 cases from 2004 to 2010. Prz Gastroenterol.

[35] Valadao M, Braggio D, Santos AF, Pimenta-Inada HK, Linhares E, Goncalves R, Romano S, Vilhena B, Small I, Cubero D, Cruz F, Oliveira AT, Martinho O (2012). Involvement of signaling molecules in the prediction of response to imatinib treatment in metastatic GIST patients. J Surg Res.

[36] Kang YN, Jung HR, Hwang I (2010). Clinicopathological and immunohistochemical features of gastointestinal stromal tumors. Cancer Res Treat.

[37] Singer S, Rubin BP, Lux ML, Chen CJ, Demetri GD, Fletcher CD, Fletcher JA (2002). Prognostic value of KIT mutation type, mitotic activity, and histologic subtype in gastrointestinal stromal tumors. J Clin Oncol.

[38] Lamba G, Gupta R, Lee B, Ambrale S, Liu D (2012). Current management and prognostic features for gastrointestinal stromal tumor (GIST). Exp Hematol Oncol.

[39] Reith JD, Goldblum JR, Lyles RH, Weiss SW (2000). Extragastrointestinal (soft tissue) stromal tumors: an analysis of 48 cases with emphasis on histologic predictors of outcome. Mod Pathol.

[40] Martín J, Poveda A, Llombart-Bosch A, Ramo s R, López-Guerrero JA, García del Muro J, Maurel J, Calabuig S, Gutierrez A, González de Sande JL, Martínez J, De Juan A, Laínez N, Spanish Group for Sarcoma Research (2005). Deletions affecting codons 557-558 of the c-KIT gene indicate a poor prognosis in patients with completely resected gastrointestinal stromal tumors: a study by the Spanish Group for Sarcoma Research (GEIS). J Clin Oncol.

[41] Gits CM, van Kuijk PF, Jonkers MB, Boersma AW, van Ijcken WF, Wozniak A, Sciot R, Rutkowski P, Schoffski P, Taguchi T, Mathijssen RH, Verweij J, Sleijfer S (2013). MiR-17-92 and miR-221/222 cluster members target KIT and ETV1 in human gastrointestinal stromal tumours. Br J Cancer.

[42] Koelz M, Lense J, Wrba F, Scheffler M, Dienes HP, Odenthal M (2011). Down-regulation of miR-221 and miR-222 correlates with pronounced Kit expression in gastrointestinal stromal tumors. Int J Oncol.

[43] Haller F, von Heydebreck A, Zhang JD, Gunawan B, Langer C, Ramadori G, Wiemann S, Sahin O (2010). Localization- and mutation-dependent microRNA (miRNA) expression signatures in gastrointestinal stromal tumours (GISTs), with a cluster of co-expressed miRNAs located at 14q32.31. J Pathol.

[44] Martin-Broto J, Gutierrez A, Garcia-del-Muro X, Lopez-Guerrero JA, Martinez-Trufero J, de Sande LM, Lainez N, Maurel J, De Juan A, Losa F, Andres R, Casado A, Tejido PG (2010). Prognostic time dependence of deletions affecting codons 557 and/or 558 of KIT gene for relapse-free survival (RFS) in localized GIST: a Spanish Group for Sarcoma Research (GEIS) Study. Ann Oncol.

[45] Liegl B, Kepten I, Le C, Zhu M, Demetri GD, Heinrich MC, Fletcher CD, Corless CL, Fletcher JA (2008). Heterogeneity of kinase inhibitor resistance mechanisms in GIST. J Pathol.

[46] Fornari F, Gramantieri L, Ferracin M, Veronese A, Sabbioni S, Calin GA, Grazi GL, Giovannini C, Croce CM, Bolondi L, Negrini M (2008). MiR-221 controls CDKN1C/p57 and CDKN1B/p27 expression in human hepatocellular carcinoma. Oncogene.

[47] le Sage C, Nagel R, Egan DA, Schrier M, Mesman E, Mangiola A, Anile C, Maira G, Mercatelli N, Ciafre SA, Farace MG, Agami R (2007). Regulation of the p27(Kip1) tumor suppressor by miR-221 and miR-222 promotes cancer cell proliferation. EMBO J.

[48] Medina R, Zaidi SK, Liu CG, Stein JL, van Wijnen AJ, Croce CM, Stein GS (2008). MicroRNAs 221 and 222 bypass quiescence and compromise cell survival. Cancer Res.

[49] Chun-Zhi Z, Lei H, An-Ling Z, Yan-Chao F, Xiao Y, Guang-Xiu W, Zhi-Fan J, Pei-Yu P, Qing-Yu Z, Chun-Sheng K (2010). MicroRNA-221 and microRNA-222 regulate gastric carcinoma cell proliferation and radioresistance by targeting PTEN. BMC Cancer.

[50] Ihle MA, Trautmann M, Kuenstlinger H, Huss S, Heydt C, Fassunke J, Wardelmann E, Bauer S, Schildhaus HU, Buettner R, Merkelbach-Bruse S (2015). miRNA-221 and miRNA-222 induce apoptosis via the KIT/AKT signalling pathway in gastrointestinal stromal tumours. Mol Oncol.

[51] Lasota J, Wozniak A, Sarlomo-Rikala M, Rys J, Kordek R, Nassar A, Sobin LH, Miettinen M (2000). Mutations in exons 9 and 13 of KIT gene are rare events in gastrointestinal stromal tumors. A study of 200 cases. Am J Pathol.

[52] Corless CL, McGreevey L, Haley A, Town A, Heinrich MC (2002). KIT mutations are common in incidental gastrointestinal stromal tumors one centimeter or less in size. Am J Pathol.

[53] Lazar AJ, Tuvin D, Hajibashi S, Habeeb S, Bolshakov S, Mayordomo-Aranda E, Warneke CL, Lopez-Terrada D, Pollock RE, Lev D (2008). Specific mutations in the beta-catenin gene (CTNNB1) correlate with local recurrence in sporadic desmoid tumors. Am J Pathol.

