# Supplementary material for: Gene expression analyses determine two different subpopulations in KIT-negative GIST-like (KNGL) patients
Source: Oncotarget. 2018 Apr 3;9(25):17576–88. doi: 10.18632/oncotarget.24799 (PMC5915139; doi:10.18632/oncotarget.24799)
Supplement: Supplementary file 1 [file oncotarget-09-17576-s001.pdf]

Gene expression analyses determine two different sub-populations in KIT-negative GIST-like (KNGL) patients

SUPPLEMENTARY MATERIALS

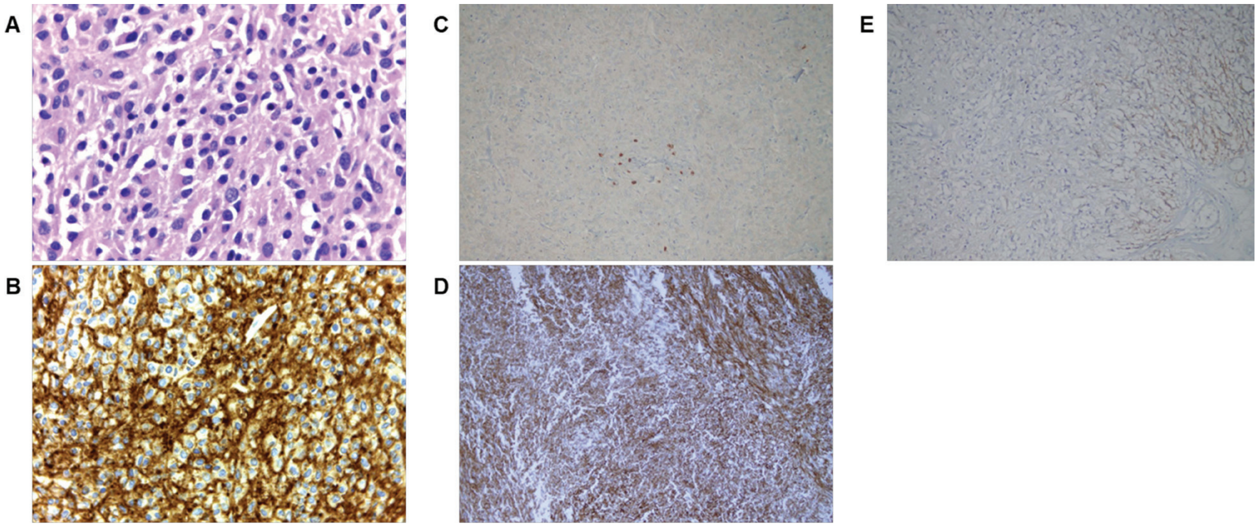

Supplementary Figure 1: Epithelioid GIST: (**A**) Tumor cells with abundant eosinophilic cytoplasm and nuclear atypia (H&E 20×). (**B**) Strong membranous and cytoplasmic immunoreactivity for DOG1 (20×). (**C**) C-Kit negative immunostaining, with mastocytes stained positively with c-Kit (10×). (**D**) GIST c-Kit positive (10×). (**E**) KNGL with less than 10% of c-Kit immunostaining (10×).

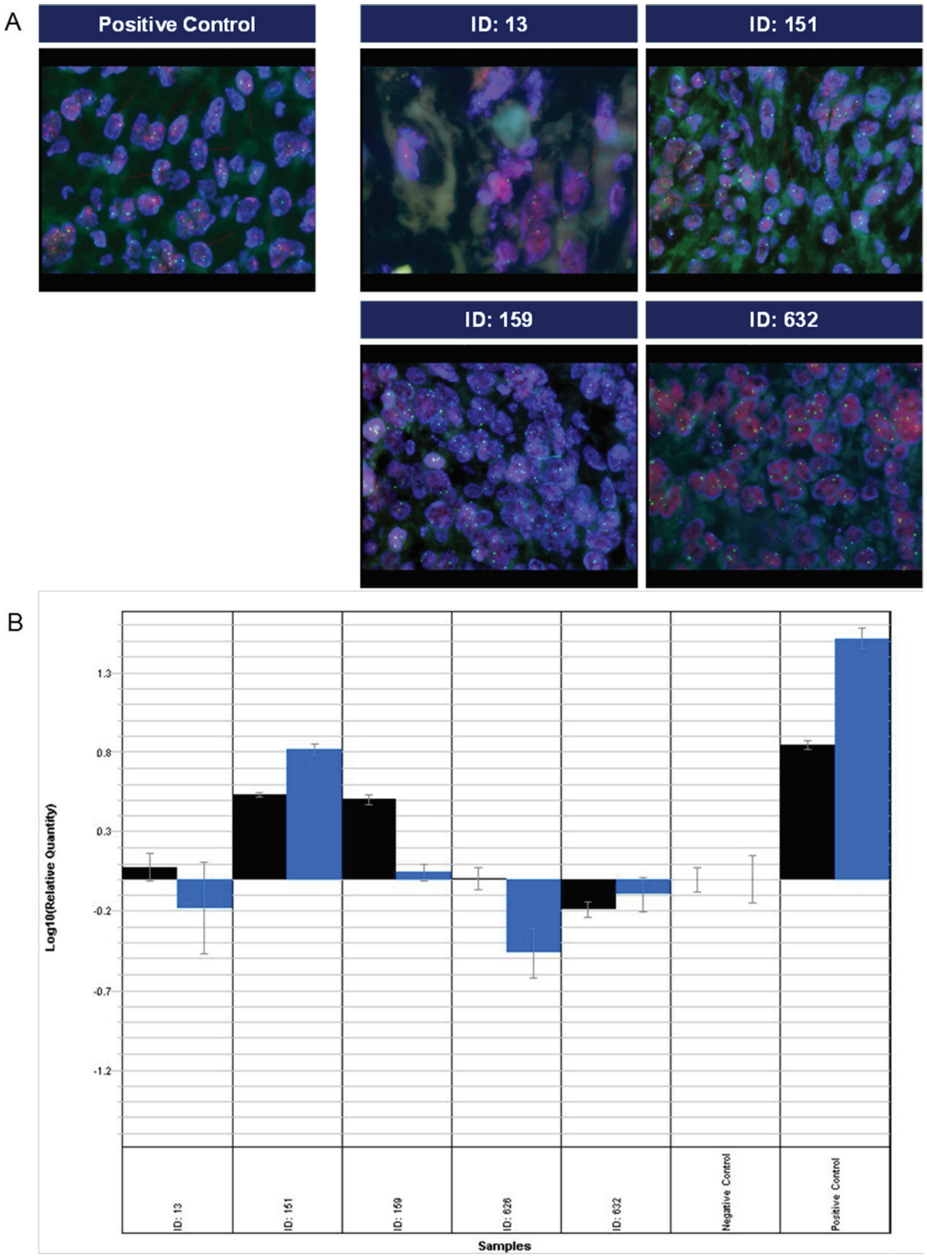

Supplementary Figure 2: (**A**) Amplification of *MDM2* was performed, by FISH in 4 KNGL cases. A dedifferentiated liposarcoma was used as positive control. Case with ID: 151 showed positive amplification of *MDM2*.(**B**) The expression levels of *CDK4* and *MDM2*, of 5 KNGL cases, were analyzed by qRT-PCR. A dedifferentiated liposarcoma was used as positive control and a KIT-positive GIST was used as negative control and reference. Moderate expression of both genes was determined in case with ID: 151.

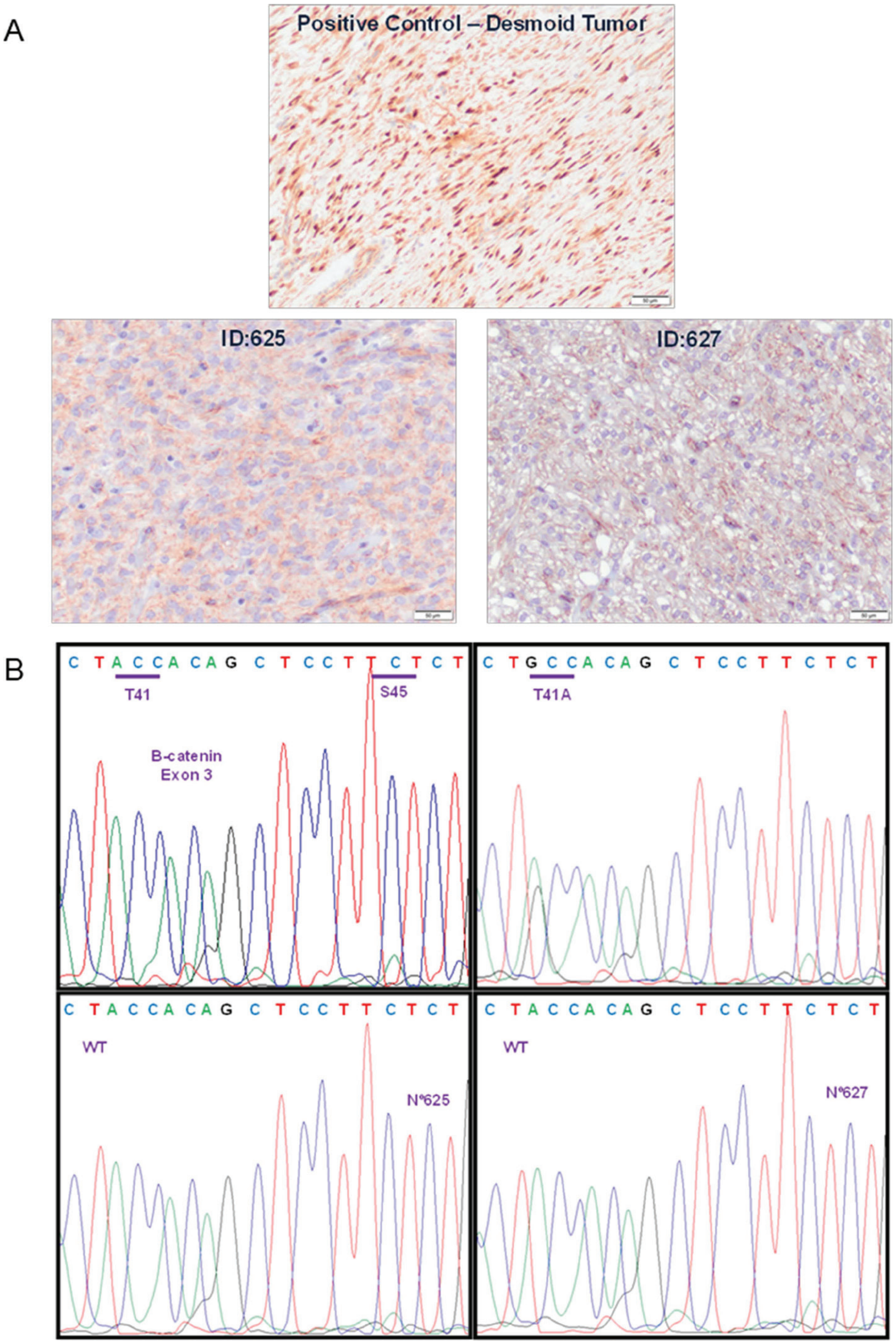

Supplementary Figure 3: (**A**) B-Catenin cytoplasmic localization by immunohistochemistry, of case 625 (down-left) and case 627 (down-right). A diagnosed desmoid tumor was used as positive control (up) – nuclear β-catenin (**B**) *CTNNB1* mutations analysis, by Sanger Sequencing, of case 625 (down-left) and case 627 (down-right). A diagnosed desmoid tumor was used as positive control (up-right). Wild type (WT) β-Catenin gene sequence is also represented (up-left).

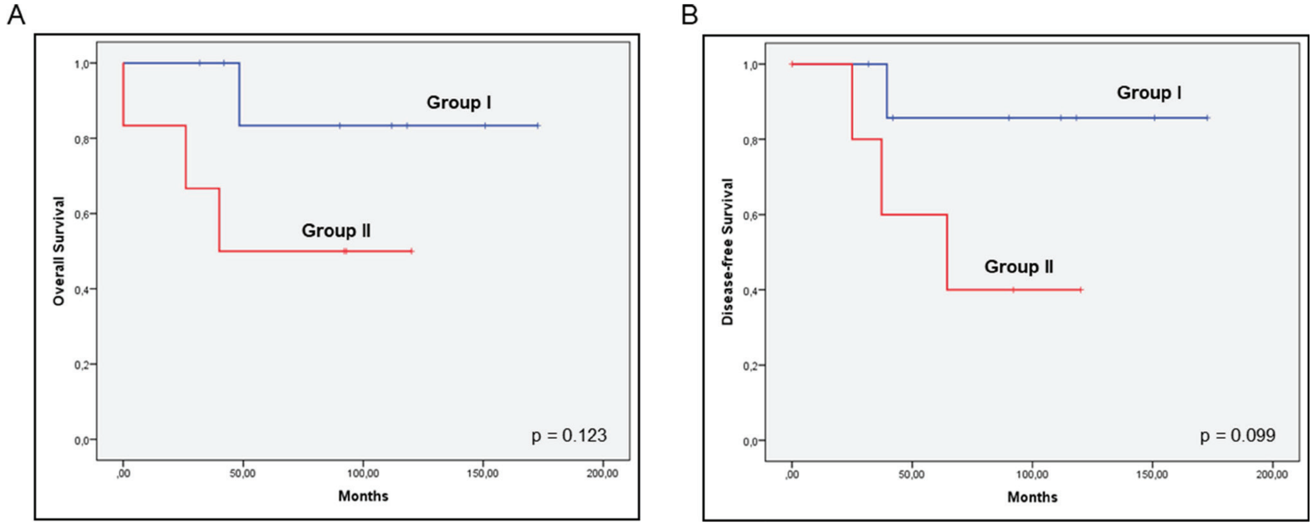

Supplementary Figure 4: Survival analysis taking into consideration only *KIT* and *PDGFRA* mutated cases. Group I (n = 8) and Group II (n = 6).

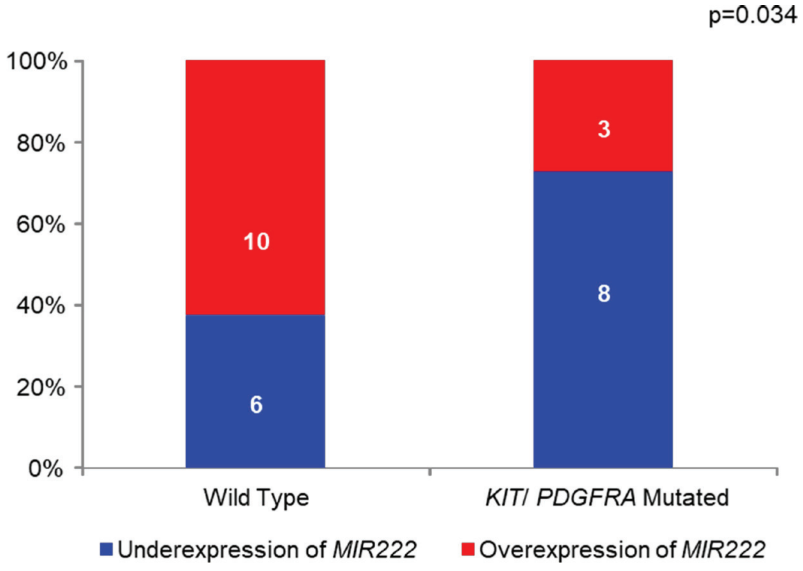

Supplementary Figure 5: Mutational analyses according to miR221-222 expression.

Supplementary Table 1: Influence of miRNA221/ 222 expression and clinical data

**Cellularity:**			0.24			0.26
Normal	6 (75%)	10 (48%)		9 (64%)	5 (38%)	
High	2 (25%)	11 (52%)		5 (36%)	8 (61%)	
**Size (cm):**			0.32			0.71
0–6	5 (62%)	10 (48%)		7 (50%)	8 (61%)	
6–10	0 (0%)	5 (24%)		4 (29%)	2 (15%)	
>10	3 (37%)	6 (29%)		3 (21%)	3 (23%)	
**Number of mitosis:**			0.044			0.13
0–10 MPF	7 (87%)	9 (43%)		10 (71%)	5 (38%)	
>10 MPF	1 (12%)	12 (57%)		4 (29%)	8 (61%)	
**Location:**			0.038			0.054
Gastric	2 (25%)	15 (71%)		5 (36%)	10 (77%)	
Others	6 (75%)	6 (29%)		9 (64%)	3 (23%)	
**Age:**			0.38			0.69
0–60 years	5 (71%)	9 (43%)		7 (54%)	5 (38%)	
>60 years	2 (29%)	12 (57%)		6 (46%)	8 (61%)	
**Diagnostic delay (months):**			0.68			0.25
0–1	5 (62%)	10 (48%)		10 (71%)	6 (46%)	
>1	3 (37%)	11 (52%)		4 (29%)	7 (54%)	
**Exon 11 mutated:**			0.12			0.22
No	4 (67%)	19 (95%)		9 (75%)	12 (100%)	
Yes	2 (33%)	1 (5%)		3 (25%)	0 (0%)	
**KIT expression:**			0.21			0.018
≤10%	4 (50%)	5 (24%)		9 (64%)	2 (15%)	
Absolute negative	4 (50%)	16 (76%)		5 (36%)	11 (85%)	
**Subtype:**			0.2			0.054
Epithelioid	5 (62%)	6 (29%)		9 (64%)	3 (23%)	
Others	3 (37%)	15 (71%)		5 (36%)	10 (77%)	
**Expression group[a]:**			0.2			0.046
I	5 (62%)	6 (29%)		8 (57%)	2 (15%)	
II	3 (37%)	15 (71%)		6 (43%)	11 (85%)	

[a]I – *KIT*/*PDGFRA*/*DOG1* positive; II – *IGF1R* positive. *N* = 27.
